# Anti-E Alloimmunization: A Rare Cause of Severe Fetal Hemolytic Disease Resulting in Pregnancy Loss

**DOI:** 10.1155/2009/471623

**Published:** 2010-03-09

**Authors:** An-Shine Chao, Angel Chao, See-Yin Ho, Yao-Lung Chang, Reyin Lien

**Affiliations:** ^1^Department of Obstetrics and Gynecology, Chang Gung Memorial Hospital, College of Medicine, Chang Gung University, Taoyuan 333, Taiwan; ^2^Department of Pediatrics, Chang Gung Memorial Hospital, College of Medicine, Chang Gung University, Taoyuan 333, Taiwan

## Abstract

We report a case of severe intrauterine hemolysis caused by sole anti-E alloimmunization. A 36-year-old multipara woman presented with hydrops fetalis at 27 weeks of gestation. She had a history of previous neonatal death. In this pregnancy, she was found to have very high titer of anti-E antibody. Ultrasonography detected marked skin edema, cardiomegaly, hepatosplenomegaly, pleural effusion, ascites, placentomegaly, and polyhydramnios. The Doppler peak systolic velocity in the middle cerebral artery was 0.8 m/s, indicating severe fetal anemia. Multiple intrauterine transfusions for the anemic fetus were administered. However, persistent severe fetal anemia and placentomegaly caused poor neonatal death and mirror syndrome in the mother. Uncommon red blood cell alloimmunization has to be watched for early in any population, especially in a woman with a history of unexplained perinatal loss.

## 1. Introduction

Antibodies with anti-E specificity are detected in 14–20% of pregnant women and it is one of the most common non-D Rhesus (Rh) antibody in the pathogenesis of neonatal hemolytic disease [1, 2]. However, anti-E is rarely associated with severe hemolytic anemia in the fetus [3, 4]. We report an unusual case of severe intrauterine hemolysis due to anti-E alloimmunization.

## 2. Case Presentation

**Figure 1:**
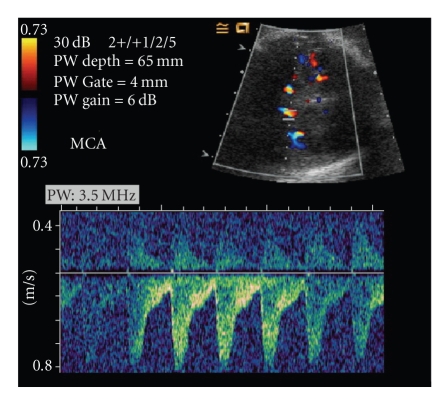
Doppler peak systolic velocity in the fetal middle cerebral artery showing severe fetal anemia.

A 36-year-old woman, G5P2AA1, was referred to our clinic at 27 weeks gestation due to the presence of hydrops fetalis. Antenatal examination at outside hospital is noncontributory for any hydrops fetalis. She had two previous cesarean sections without history of blood transfusion. The first baby was a term live-born. The second baby was born at 36 weeks gestation died in a few days after birth of unknown neonatal hepatomegaly only 3 years ago. Sonography of the fetus revealed marked skin edema, cardiomegaly, hepatosplenomegaly, pleural effusion, ascites, placentomegaly, and polyhydramnios. Doppler peak systolic velocity (PSV) in the middle cerebral artery (MCA) was 0.8 m/s (>2.0 multiples of median) (MoM), indicating severe fetal anemia (Figure 1). Studies on karyotype, thalassemia, Parvovirus B19, cytomegalovirus, Toxoplasmosis, and antinuclear antibodies were all normal. Both parents had O and D+ blood type. Indirect Coombs' test was positive and high titer of anti-E antibodies with antihuman globulin titers of 1  :  1024 was noted in the maternal blood. Rh typing of the mother was D(+) E(−) c(−) C(+) e(+). Severe fetal anemia was confirmed by umbilical cord blood sampling with hemoglobin (Hb) level of 2.1 g/dL and hematocrit of 6.1%. The phenotype of fetal red blood cells (RBCs) was O, D (+) E(+) c(+) C(+) e(+) and strong positive in direct Coombs' test (++++). Three times of 20 mL each in every week antenatal transfusions of irradiated Group O, D+ packed RBCs were administered between 28 and 30 weeks of gestation. Fetal anemia persisted between Hb levels between 3-4 g/dL with no improvement of hydrops and Doppler flow studies. She developed preeclampsia with elevated blood pressure and general edema. Cesarean section was performed at 31 weeks gestation after steroid administration for the mother who had twice section, delivering a 2000 g male baby with Apgar scores of 2 and 3 at 1 and 5 minutes, respectively. The placenta was bulky and grossly edematous weighing 1300 g. Even with multiple blood transfusions, the newborn succumbed in 2 days. Autopsy was declined.

## 3. Discussion

Anti-E alloimmunization can cause fetal anemia, and the incidence could be underestimated [5]. Only a few reports of pregnancy loss due to anti-E were described [4, 6]. Unlike anti-D alloimmunization, anti-E titer is less sensitive in detecting severity of hemolysis in the subsequent pregnancy. Therefore, high level of suspicion and early recognition of these cases is crucial even with low titers. A critical titer of 1  :  16 has been considered in anti-E alloimmunization [4]. Joy et al. reported that six of the 16 fetuses with maternal titer greater than 1  :  16 had fetal Hb less than 10 g/dL. Two fetal hydrops resulted in perinatal death despite intrauterine treatment. Extremely elevated titer coupled with very severe fetal anemia as in this present study is scarce [4, 6]. 

Using a cut-off of 1.5 MoM in the MCA of PSV Doppler studies, 100% sensitivity in the prediction of moderate to severe fetal anemia was reported [7]. If MCA PSV is added, fetal anemia is treatable with favorable outcome of over 90% before hydrops [8]. Fetal hydrops combined with very low cord blood Hb (2.1 g/dL) is a late and ominous presentation of hemolytic disease. Encountering this adverse clinical setting, receiving blood transfusions in our case was in vain.

An uncommon consequence of fetal hydrops that will worsen the condition is the development of maternal mirror syndrome. The trigger of rapid body weight gain, hydramnios, elevated blood pressure, and general edema in mirror syndrome may be derived from a compromised fetus or placenta. Immediate delivery should be performed to avoid maternal morbidity. 

In conclusion, we advocate that in D+ women who had previous unexplained fetal loss (fetal hydrops), screening of maternal serum alloantibodies against RBCs is important in the early trimesters of pregnancy, followed by regular Doppler studies and treatment strategies if alloimmune cause of hemolysis is identified.
